# Prognostic factors after pulmonary metastasectomy of colorectal cancers: influence of liver metastasis

**DOI:** 10.1186/s12957-016-0940-3

**Published:** 2016-07-29

**Authors:** Seok Kim, Hong Kwan Kim, Jong Ho Cho, Yong Soo Choi, Kwhanmien Kim, Jhingook Kim, Jae Ill Zo, Young Mog Shim, Jin Seok Heo, Woo Yong Lee, Hee Cheol Kim

**Affiliations:** 1 Department of Thoracic and Cardiovascular Surgery; Sungkyunkwan University School of Medicine, Samsung Medical Center, Seoul, 06351 South Korea; 2 Department of Thoracic and Cardiovascular Surgery, Seoul National University Bundang Hospital, Seoul National University College of Medicine, Gyeonggi-do, 13620 South Korea; 3 Department of Surgery, Samsung Medical Center, Sungkyunkwan University School of Medicine, 81 Irwon-ro, Gangnam-gu, Seoul 06351 South Korea

**Keywords:** Colorectal cancer, Metastasectomy, Pulmonary metastasectomy, Liver metastasis, Disease-free survival

## Abstract

**Background:**

Our objective was to evaluate the influence of liver metastasis on survival after pulmonary metastasectomy in patients with colorectal cancer (CRC).

**Methods:**

We retrospectively reviewed a total of 524 patients and were classified into two groups based on the presence of liver metastasis. Group HM + PM (*n* = 106) included patients who previously received a hepatic metastasectomy and then received pulmonary metastasectomy. Group PM (*n* = 418) included patients who only received pulmonary metastasectomy with no liver metastasis.

**Results:**

There were more male patients (70 vs. 57 %; *P* = 0.02) and more patients with colon cancer (60 vs. 42 %, *P* = 0.001) in group HM + PM than in group PM. Otherwise, there was no significant difference between the two groups in clinicopathologic characteristics and extent of surgery. The 5-year overall survival (OS) and disease-free survival (DFS) rates were 58 and 31 %, respectively. There was no significant difference in OS (group HM + PM, 54 % vs. group PM, 59 %; *P* = 0.085) and in DFS (group HM + PM, 28 % vs. group PM, 32 %; *P* = 0.12). For the entire patient cohort, a multivariate analysis revealed that the presence of liver metastasis, CRC T and N stages, disease-free interval, and number and size of lung metastases were significantly associated with OS and DFS.

**Conclusions:**

Our findings suggest that previous or present liver metastasis should not exclude a patient from pulmonary metastasectomy. When lung metastasis is detected in patients with a history of hepatic metastasectomy, pulmonary metastasectomy is still a viable treatment option especially in patients with a long disease-free interval and a small number of lung metastases.

## Background

Lung metastasis in patients with colorectal cancer (CRC) suggests the possibility of widespread hematogenous dissemination of cancer, which needs to be treated with systemic chemotherapy [1]. However, unsatisfactory outcomes of chemotherapy for CRC patients with lung metastases have provided a basis for surgical resection of lung metastases [2]. Despite doubts about the role of local treatment for systemic metastasis, many surgeons have performed pulmonary metastasectomy in properly selected patients with reported 5-year survival rates ranging from 27 to 68 % [3–6].

However, it is controversial whether pulmonary metastasectomy is still indicated for patients with a history of hepatic metastasectomy. It also remains unknown whether patients undergoing both hepatic and pulmonary metastasectomies have comparable survival outcomes to those undergoing pulmonary metastasectomy only. Some authors demonstrated that a previous hepatic metastasectomy increased the risk of death in patients undergoing pulmonary metastasectomy [7–10], whereas most studies have reported that a history of liver metastasis at the time of pulmonary metastasectomy is not an adverse factor impairing survival [6, 8, 11–17]. Therefore, it should be noted that confining the candidates for metastasectomy to patients with single-organ metastasis might deny some patients a chance for long-term survival.

In our institution, we aggressively tried to perform pulmonary metastasectomy even in patients with a history of liver metastases as long as those candidates fulfill the criteria for pulmonary metastasectomy and liver metastases had been completely resected. The objective of this study was to evaluate whether a history of hepatic metastasectomy influences survival in CRC patients undergoing pulmonary metastasectomy by comparing treatment outcomes between patients with and without a history of hepatic metastasectomy. In addition, we performed prognostic factor analyses in order to find which factors affected survival.

## Methods

Between January 1994 and December 2012, a total of 532 consecutive patients underwent pulmonary metastasectomy for lung metastases of CRC at our institution. Among these, 106 patients underwent metastasectomy for lung metastases detected during follow-up after successful hepatic metastasectomy (*n* = 102) or underwent both hepatic and pulmonary metastasectomies simultaneously (*n* = 4) (group HM + PM). The remaining 418 patients received pulmonary metastasectomy only without a history of extrapulmonary metastasis or liver metastases (group PM). Patients were excluded if they had metastasectomy for liver metastases detected during follow-up after successful pulmonary metastasectomy (*n* = 8). The indication of pulmonary metastasectomy were (1) control of the primary CRC, (2) no extrapulmonary metastases, (3) completely resectable lung metastases at preoperative imaging studies, and (4) sufficient cardiopulmonary reserve for pulmonary metastasectomy. To ensure that the indication of pulmonary metastasectomy was fulfilled, patients need to undergo chest computed tomography (CT) and abdomen CT and/or positron emission tomography scan preoperatively. Their medical records were retrospectively reviewed to assess clinical characteristics, early postoperative outcomes, recurrence pattern, and survival. This study was reviewed and approved by the Institutional Review Board of Samsung Medical Center.

After pulmonary metastasectomy, patients were regularly evaluated by chest CT every 3 to 4 months for the first 2 years following surgery and then every 6 months thereafter. Serum carcinoembryonic antigen (CEA) level was routinely checked at every outpatient visit. When the extrapulmonary recurrence was suspected, we tried to obtain histological or unequivocal radiological proof. If pulmonary recurrence was considered resectable, aggressive surgical resection was carried out.

Descriptive statistics were used to assess patient demographic characteristics and outcomes. Normally distributed continuous data were expressed as means ± standard deviations. Categorical data were expressed as counts and proportions. Student’s *t* tests or Wilcoxon rank-sum test, depending on the normality of distribution, and the *χ*^2^ test or Fisher’s exact tests were used to compare continuous and categorical variables, respectively. One-way analysis of variance or Kruskal-Wallis test, depending on the normality of distribution, was used to compare the continuous variables among three groups. The disease-free interval (DFI) was calculated as the interval between the date of CRC resection and the date of pulmonary metastasectomy. Overall survival (OS) was defined as the time from the date of pulmonary metastasectomy until the last date of follow-up for patients who remained alive or until death. Disease-free survival (DFS) was defined as the time from the date of pulmonary metastasectomy to recurrence or death. Survival curves were prepared using the Kaplan-Meier method and were compared univariately using the log-rank test. All statistical tests were two-sided with a significance level set at 0.05 and were performed using Stata software version 10.0 (Stata, College Station, TX, USA).

## Results

### Patient characteristics

Patient characteristics are summarized in Table 1. The study included 314 men (60 %) and 210 women (40 %) with a mean age of 59 years (range, 30 to 83 years). There were more male patients in group HM + PM than in group PM (70 vs. 57 %, *P* = 0.02). With regard to the location of primary tumor, more patients in group HM + PM had colon cancer (60 vs. 42 %, *P* = 0.001) compared with group PM in which there were more patients with rectal cancer. Otherwise, there was no statistically significant difference between the two groups in terms of age at the time of pulmonary metastasectomy, pathologic stage of primary tumor, number of lung metastases, size of the largest lung metastasis, extent of pulmonary resection, thoracoscopic surgery, and adjuvant chemotherapy. Details of surgical techniques are shown in Table 2.

**Table 1:** Patients characteristics

	All patients (*n* = 524)	Group HM + PM (*n* = 106)	Group PM (*n* = 418)	*P* value
	No. (%)	No. (%)	No. (%)	
Age at PM (year, mean)	59.2 (30–83)	59.8 (30–83)	59.1 (31–82)	0.427
Sex				0.02
Male	314 (40.1)	74 (30.2)	240 (57.4)	
Female	210 (59.9)	32 (69.8)	178 (42.6)	
Location of primary tumor				0.001
Colon	239 (45.6)	64 (60.4)	175 (41.9)	
Rectum	285 (54.4)	42 (39.6)	243 (58.1)	
T stage of CRC				0.703
pT1	11 (2.1)	2 (1.9)	9 (2.2)	
pT2	45 (8.6)	7 (6.6)	38 (9.1)	
pT3	345 (65.8)	75 (70.8)	270 (64.6)	
pT4	123 (23.5)	22 (20.8)	101 (24.2)	
N stage of CRC				0.839
pN0	155 (29.6)	34 (32.1)	121 (28.9)	
pN1	204 (38.9)	39 (36.8)	165 (39.5)	
pN2	164 (31.3)	33 (31.1)	131 (31.3)	
pN3	1 (0.2)	0 (0)	1 (0.2)	
Disease-free interval^a^				0.927
<12 months	112 (21.4)	23 (21.7)	89 (21.3)	
≥12 months	412 (78.6)	83 (78.3)	329 (78.7)	
Location of lung metastasis				0.787
Unilateral	425 (81.1)	85 (80.2)	340 (81.3)	
Bilateral	99 (18.9)	21 (19.8)	78 (18.7)	
Number of lung metastasis				0.289
Single	349 (66.6)	66 (62.3)	283 (67.7)	
Multiple	175 (33.4)	40 (37.7)	135 (32.3)	
Adjuvant chemotherapy	376 (71.8)	83 (78.3)	293 (70.1)	0.094

*PM* pulmonary metastasectomy, *CRC* colorectal cancer

^a^Disease-free interval was calculated as the interval between the date of colorectal cancer resection and the date of pulmonary metastasectomy

**Table 2:** Details of surgical techniques

	All patients (*n* = 524)	Group HM + PM (*n* = 106)	Group PM (*n* = 418)	*P* value
	No. (%)	No. (%)	No. (%)	
Extent of PM				0.35
Precision excision	20 (3.8)	5 (4.7)	15 (3.6)	
Wedge resection	368 (70.2)	77 (72.6)	291 (69.6)	
Segmentectomy	37 (7.1)	4 (3.8)	33 (7.9)	
Lobectomy	93 (17.8)	18 (17)	75 (17.9)	
Bilobectomy	4 (0.8)	1 (0.9)	3 (0.7)	
Sleeve resection^a^	2 (0.4)	1 (0.9)	1 (0.2)	
Extent of HM				
Chemotherapy		6 (5.7)		
RFA or TACE		23 (21.7)		
Wedge resection or tumorectomy		11 (10.4)		
Segmentectomy		26 (24.5)		
Lobectomy		32 (30.2)		
Extended hemihepatectomy		8 (7.5)		
Thoracoscopic surgery	298 (56.9)	61 (57.6)	237 (56.7)	0.875
Mediastinal LND	140 (26.7)	28 (26.4)	112 (26.8)	0.937

*PM* pulmonary metastasectomy, *HM* hepatic metastasectomy, *RFA* radiofrequency ablation, *TACE* transarterial chemoembolization, *LND* lymph node dissection

^a^Sleeve resection included one sleeve lobectomy and one sleeve bilobectomy

### Treatment outcomes

Postoperative complications occurred in 22 patients (4.2 %), including pneumothorax in six patients, arrhythmia in six, and prolonged air leak in six. Patients in group HM + PM (7.6 %) experienced complications more frequently than in group PM (3.4 %), but there was no statistically significant difference (*P* = 0.098). In-hospital mortality occurred in one patient from group HM + PM (0.19 %) and the cause of death was acute respiratory distress syndrome.

**Fig. 1:**
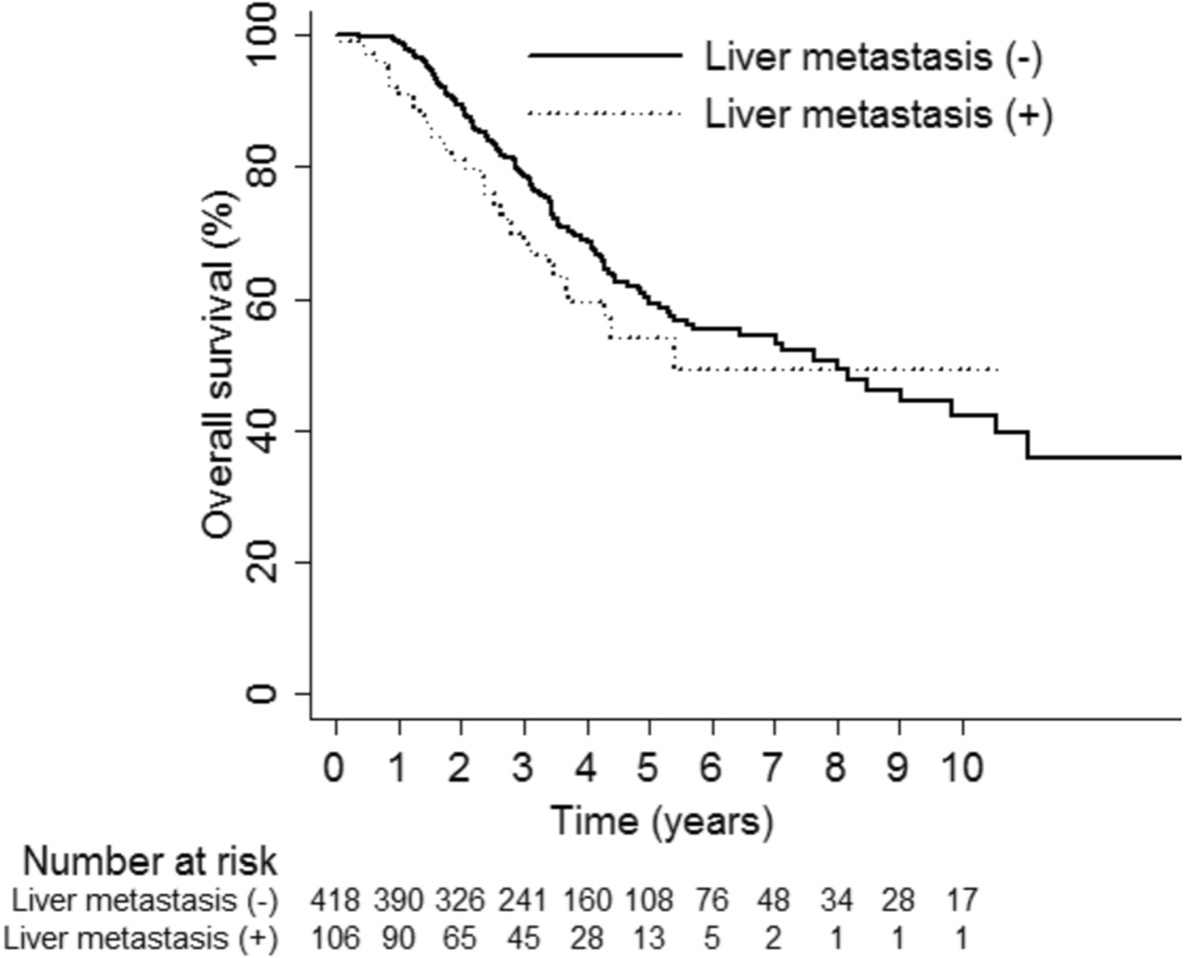
Comparison of overall survival between group HM + PM and group PM

**Fig. 2:**
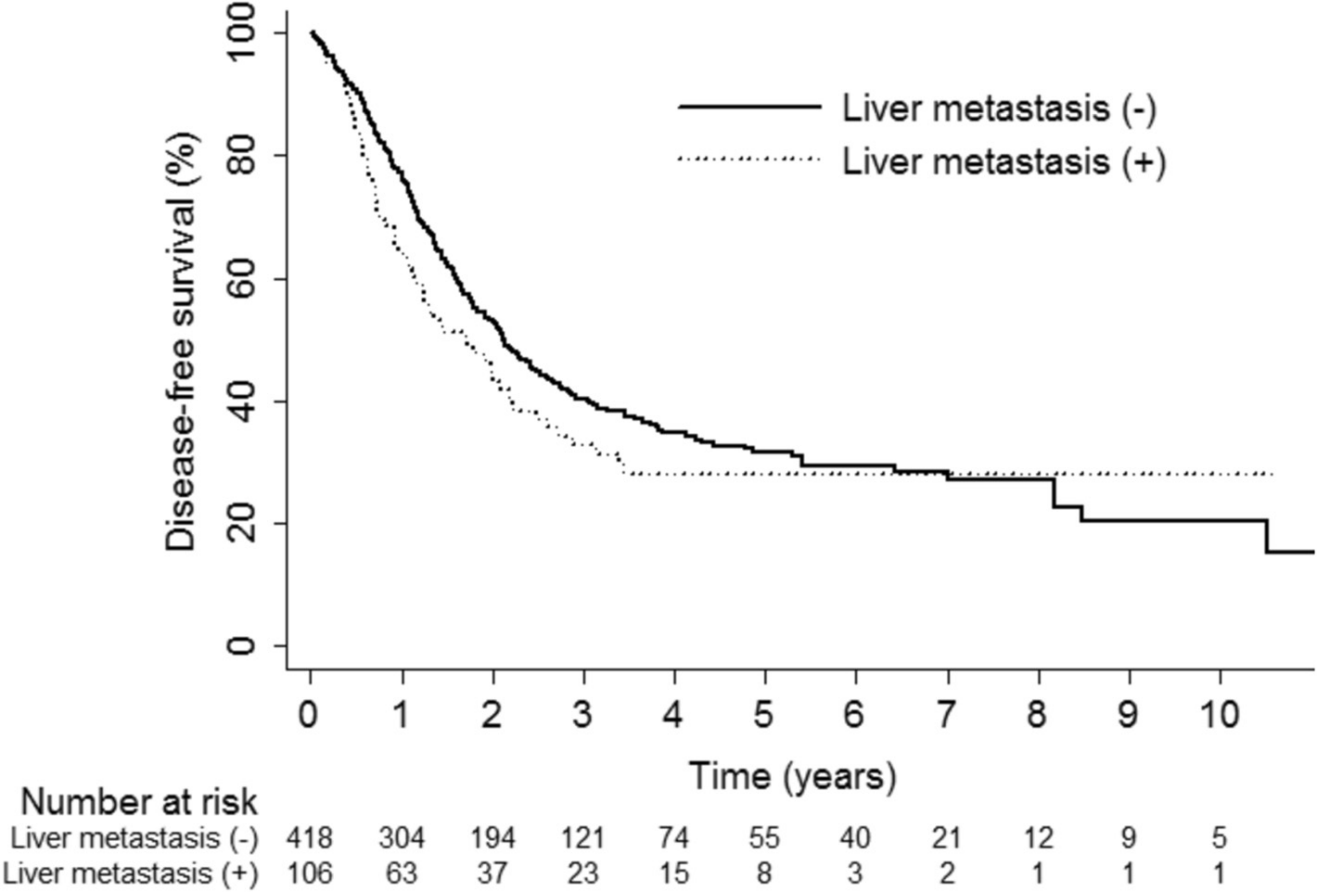
Comparison of disease-free survival between group HM + PM and group PM

The mean follow-up duration was 45 months (0.2 to 217 months). At the end of follow-up, 176 of 524 patients (34 %) had died, and the remaining 348 patients (66 %) were alive. The median OS time was 91.5 months (95 % confidence interval (CI) 64.9~126.3), and OS rate at 5 years was 58 %. There was no significant difference in the 5-year OS rates between the two groups (group HM + PM, 54 % vs. group PM, 59 %; *P* = 0.085; Fig. 1). During follow-up, 286 patients (55 %) developed recurrence and of these, 248 (86.7 %) had distant metastasis. Of these, 41 patients underwent repeated pulmonary metastasectomy for recurrent lung metastases since they fulfilled the criteria of pulmonary metastasectomy. Data regarding recurrence pattern are summarized in Table 3. The median DFS time was 24.9 months (95 % CI 21.2~27.5), and DFS rate at 5 years was 31 %. There was no significant difference in the 5-year DFS rates between the two groups (group HM + PM, 28 % vs. group PM, 32 %; *P* = 0.12; Fig. 2).

**Table 3:** Summary of recurrence in the study population

	Number of patients (%)			
	All patients (*n* = 524)	Group HM + PM (*n* = 106)	Group PM (*n* = 418)	*P* value
Overall recurrence	286 (54.6)	58 (54.7)	228 (54.6)	0.975
Locoregional	20 (3.8)	4 (3.8)	16 (3.8)	0.979
Distant	248 (47.3)	52 (49.1)	196 (46.9)	0.69
Both	18 (3.6)	2 (2)	16 (4)	0.326

### Prognostic factor analysis

To better understand the factors associated with improved outcomes, univariate and multivariate analyses were performed and detailed in Tables 4 and 5. For the entire patient population, history of hepatic metastasectomy before pulmonary metastasectomy, advanced pathologic T and N stage of primary CRC, DFI (<12 months), number of lung metastases, and size of the largest lung metastasis were independent prognostic factors associated with worse OS and DFS (Table 4). When a prognostic factor analysis was confined to patients who had a history of previous hepatic metastasectomy (group HM + PM), DFI (<12 months) and number of lung metastases were independent prognostic factors associated with worse OS and DFS (Table 5).

**Table 4:** Factors associated with overall survival and disease-free survival on multivariate analyses for the entire patient population

Variable	Overall survival			Disease-free survival		
	HR	95 % CI	*P* value	HR	95 % CI	*P* value
Age at PM	1.03	1.01–1.04	0.001	–	–	–
CRC pTstage	1.65	1.23–2.2	0.001	1.22	1.0–1.48	0.05
CRC pNstage	1.32	1.08–1.61	0.006	1.19	1.03–1.38	0.021
Previous HM	1.79	1.22–2.63	0.003	1.45	1.1–1.92	0.008
DFI (≥12months)^a^	0.68	0.47–0.94	0.02	0.63	0.49–0.81	<0.001
Multiple LM	2.28	1.6–3.24	<0.001	1.66	1.27–2.16	<0.001
Size of largest LM^b^	1.06	0.94–1.19	0.343	1.1	1.0–1.2	0.043
Non-anatomical PM^c^	0.63	0.45–0.89	0.009	–	–	–
VATS	0.82	0.6–1.14	0.24	0.67	0.53–0.85	0.001
Adjuvant CTx	0.58	0.42–0.82	0.002	–	–	–

*HR* hazard ratio, *CI* confidence interval, *PM* pulmonary metastasectomy, *CRC* colorectal cancer, *HM* hepatic metastasectomy, *pTstage* pathologic T stage, *pNstage* pathologic N stage, *DFI* disease-free interval, *LM* lung metastases, *VATS* video-assisted thoracoscopic surgery, *CTx* chemotherapy

^a^Disease-free interval was calculated as the interval between the date of colorectal cancer resection and the date of pulmonary metastasectomy

^b^The long-axis diameter of the largest lung metastatic nodule was measured

^c^Non-anatomical pulmonary resection included precision excision of metastatic nodules or wedge resection

**Table 5:** Factors associated with overall survival and disease-free survival on multivariate analyses for group HM + PM

Variable	Overall survival			Disease-free survival		
	HR	95 % CI	*P* value	HR	95 % CI	*P* value
Age at PM	3.77	1.48–9.59	0.005	1.31	0.68–2.52	0.44
DFI (≥12months)^a^	0.98	0.95–0.99	0.036	0.98	0.96–0.99	0.003
Multiple LM	3.97	1.53–10.4	0.005	2.0	1.04–3.87	0.039
Size of largest LM^b^	1.69	1.00–2.83	0.048	1.2	0.89–1.63	0.227

*HR* hazard ratio, *CI* confidence interval, *PM* pulmonary metastasectomy, *DFI* disease-free interval, *LM* lung metastases

^a^Disease-free interval was calculated as the interval between the date of colorectal cancer resection and the date of pulmonary metastasectomy

^b^The long-axis diameter of the largest lung metastatic nodule was measured

## Discussion

One of the most important prerequisites is that there should be no extrapulmonary metastasis when considering pulmonary metastasectomy in CRC patients with lung metastases [3]. The presence of liver metastasis in addition to lung metastasis increases the probability of widespread hematogenous tumor dissemination, which may obviate the role of metastasectomy in patients with lung metastases. However, when patients with a history of completely resected liver metastases are diagnosed with lung metastases, it remains unknown if they would benefit from pulmonary metastasectomy or not. Is pulmonary metastasectomy justified in these patients? In order to answer this question, we conducted this study comparing survival between patients with a history of hepatic metastasectomy (group HM + PM) and those without (group PM). By this comparative analysis, we tried to evaluate whether a history of hepatic metastasectomy influences survival in CRC patients undergoing pulmonary metastasectomy. In the present study, there was no significant difference in the 5-year OS and DFS rates between the two groups. The two groups are comparable in all aspects except for gender and the location of CRC. Since there was no significant relationship between gender or the location of CRC and the survival rate in the previous literature [18], our results suggest that survival outcomes after pulmonary metastasectomy were not compromised by a history of successful hepatic metastasectomy.

Our findings are in line with previous studies regarding the outcomes of combined hepatic and pulmonary metastasectomies. Although some authors found that a previous hepatic metastasectomy was associated with poor survival in patients undergoing pulmonary metastasectomy [7–10], most studies have reported that a history of liver metastases at the time of pulmonary metastasectomy was not a significant risk factor affecting survival, and that outcomes after combined hepatic and pulmonary metastasectomies were similar to those after hepatic metastasectomy alone or pulmonary metastasectomy alone [6, 8, 11–17]. The reported 5-year OS rates ranged from 11 to 61 % after combined hepatic and pulmonary metastasectomies [8, 11–17]. Ike et al*.* showed that there was no significant difference in survival rates between patients who underwent sequential hepatic and pulmonary metastasectomies and those who underwent pulmonary metastasectomy alone [16].

However, the role of metastasectomy in patients with stage IV CRC are still controversial because these encouraging outcomes are not based on prospective randomized controlled trials comparing metastasectomy with either medical treatment or observation alone [19]. Previously reported studies might have only included candidates regarded as suitable for pulmonary metastasectomy and excluded those who are not fit for pulmonary metastasectomy [20]. In this context, the encouraging survival rate of patients undergoing hepatic metastasectomy and pulmonary metastasectomy in the present study might be attributed to the fact that selected candidates have favorable prognostic factors rather than the benefit from metastasectomy per se. Therefore, it is difficult to prove whether our good results after combined hepatic and pulmonary metastasectomies are due to metastasectomy or selection bias.

Nonetheless, the fact that the beneficial effect of pulmonary metastasectomy in patients with a previous hepatic metastasectomy has not been proved by randomized studies does not necessarily mean that the opportunity of surgery should be denied for patients who could have benefitted from pulmonary metastasectomy. For patients with liver and lung metastases that cannot be satisfactorily managed by palliative chemotherapy, it would be much more helpful to find out which patient is most likely to benefit from pulmonary metastasectomy. In previous studies, many authors tried to elucidate various prognostic factors in patients undergoing combined hepatic and pulmonary metastasectomies, including number of metastatic lesions, DFI, pretreatment CEA level, and mediastinal lymph node involvement [6–17]. In the present study, a prognostic factor analysis confined to group HM + PM revealed that shorter DFI (<12 months) and number of lung metastases were prognostic factors associated with worse OS and DFS. This means that patients who have longer DFI and small number of lung metastases are more likely to benefit from pulmonary metastasectomy even though they had a previous hepatic metastasectomy, and surgical resection for lung metastases can be more aggressively recommended for those patients.

Our study has several limitations. First, this is a retrospective study, which has many intrinsic drawbacks. Second, as discussed above, this is a nonrandomized study subject to selection bias. We should be cautious in interpreting our results because our patients selected for metastasectomy might have had inherent favorable prognostic features. The encouraging survival after pulmonary metastasectomy in overall patients and comparable outcomes between the two groups might have come from selection bias, not from the effect of treatment per se. These limitations need to be overcome by prospective randomized trial. Third, there was a significant difference in the number of patients between the two groups. This difference should be considered when interpreting our results from a statistical standpoint. This may also be related to the fact that the number of patients at risk after 4 or 5 years in group HM + PM was relatively small compared with group PM, which limits the validity of long-term survival comparison.

## Conclusions

In conclusion, we retrospectively evaluated whether a history of hepatic metastasectomy influences survival in CRC patients undergoing pulmonary metastasectomy by comparing treatment outcomes between patients with a history of hepatic metastasectomy and those without. We found that the long-term OS and DFS rates did not differ between the two groups. Given that the two groups are comparable, our findings suggest that a history of successful hepatic metastasectomy does not compromise survival in CRC patients undergoing pulmonary metastasectomy. Therefore, we believe that as long as liver metastasis is completely resected, there is no reason to deny pulmonary metastasectomy when patients have no unfavorable prognostic factors other than a history of previous hepatic metastasectomy. Based on prognostic factor analyses, patients who have longer DFI and small number of lung metastases are more likely to benefit from pulmonary metastasectomy even after hepatic metastasectomy.

## Abbreviations

CEA, carcinoembryonic antigen; CI, confidence interval; CRC, colorectal cancer; CT, computed tomography; DFI, disease-free interval; DFS, disease-free survival; HM, hepatic metastasectomy; OS, overall survival; PM, pulmonary metastasectomy
